# Expression and clinical implications of HLA-G and PD-L1 following kidney transplantation: A cohort study

**DOI:** 10.1097/MD.0000000000036053

**Published:** 2023-11-17

**Authors:** Silvia M. Botelho, Isabela J. Wastowski, Renata T. Simões, Maria A. P. C. Cysneiros, Antonio da Silva Menezes, Aline L. Rezende, Nílzio A. da Silva

**Affiliations:** a Stricto Sensu Graduate Program in Health Sciences, School of Medicine, Federal University of Goiás (UFG), Goiânia, Brazil; b State University of Goiás (UEG), Goiânia, Brazil; c Stricto Sensu Graduate Program, Faculdade Santa Casa de Belo Horizonte (FSCBH), Belo Horizonte, Brazil; d Faculty of Medicine of the Federal University of Goiás (UFG), Goiânia, Brazil.

**Keywords:** HLA-G, kidney transplantation, PD-L1, rejection

## Abstract

Kidney transplantation (KT) is the preferred treatment for end-stage renal diseases. Human leukocyte antigen G (HLA-G) and programmed death-ligand 1 (PD-L1) have notable clinical and therapeutic significance in transplantation because of their roles in promoting tolerance. This study aimed to assess HLA-G and PD-L1 levels at various stages following KT. A cohort of 12 patients was monitored from the pretransplant phase to 12 months post-surgery. Blood samples were taken at specific intervals: before kidney transplantation (T0), and then on the 7^th^ (T7), 30^th^ (T30), 90^th^ (T90), 180^th^ (T180), and 365^th^ days post transplantation. Renal biopsies were performed in patients with graft dysfunction. Plasma levels of soluble HLA-G (sHLA-G) and PD-L1 were quantified using enzyme-linked immunosorbent assays. Additionally, immunohistochemistry was used to detect the presence of both molecules in biopsy samples. Multivariate analysis indicated that episodes of rejection were correlated with decreased expression of sHLA-G (*P* < .001) and PD-L1 (*P* < .001). Over the course of the study, the sHLA-G levels also declined (*P* < .001). Patients who had been transfused had lower PD-L1 levels (*P* = .03). Furthermore, kidney recipients from related live donors had increased HLA-G expression (*P* < .001). Our findings suggest that diminished HLA-G and PD-L1 levels correlate with an increased risk of graft rejection. Notably, HLA-G expression significantly decrease after the third-month posttransplantation.

## 1. Introduction

Kidney transplantation (KT) is the optimal treatment for patients with end-stage chronic kidney disease. It not only considerably enhances patients’ quality of life and survival but is also the most cost-effective option.^[1]^ However, the primary constraint of transplantation is the immune response of the recipient against donor tissues. Irrespective of its histological type, the rejection process is influenced by various factors, each of which has a direct bearing on the long-term prognosis of the graft. Notably, the level of compatibility, as determined by human leukocyte antigen (HLA), is significant in this context.^[2]^

HLAs are pivotal molecules involved in cellular and humoral immune responses. While the polymorphic classical HLA class I (HLA-A, -B, and -C) and class II (HLA-DP, HLA-DR, and HLA-DQ) molecules instigate cellular and humoral immune responses, the non-classical HLA class I Human leucocyte antigen-G (HLA-G) mitigates these reactions.^[3]^

HLA-G has garnered attention across numerous studies owing to its tolerance-inducing role in both physiological scenarios and disease states. In contrast to classical HLA molecules, HLA-G exhibits limited genetic polymorphism, undergoes consistent alternative recombination of the primary transcript, and protein expression is largely confined to specific tissues, such as the placenta (invasive cytotrophoblasts and amniotic epithelial cells), some adult tissues (thymus, pancreas, proximal nail matrix, and cornea), and erythroblasts, and plays a key role in immune tolerance.^[4]^

Regarded as an immunological checkpoint molecule, HLA-G has become a recognized marker of immunotolerance.^[5]^ Its expression is associated with various diseases, including autoimmune disorders and transplantation.^[6]^

Another notable checkpoint molecule is programmed death-1 (PD-1) and its associated ligand, programmed death 1 ligand (PD-L1). Initially characterized as an immune inhibitory receptor on activated T and B lymphocytes and myeloid cells,^[7–10]^ subsequent research revealed that PD-1/PD-L1 binding curtails lymphocyte proliferation, thus fostering tolerance.^[11]^ The co-stimulatory pathway of PD-L1/PD1 co-stimulatory pathway plays a crucial role in suppressing alloimmune responses and instigating and sustaining peripheral tolerance. The expression of PD-L1, both in tissues and within lymphocytes, might be instrumental in graft tolerance.^[12]^ Multiple studies have implied that PD-1 exerts a significant immunoregulatory influence on the allograft response, positioning this molecule as a prospective therapeutic target in transplantation.^[13,14]^

Given this backdrop, our study aimed to assess HLA-G and PD-L1 levels before and after KT over a 12-month follow-up period. We then correlated these findings with clinical and laboratory metrics related to allograft outcomes.

## 2. Materials and methods

### 2.1. Ethical considerations

This study was approved by the Research Ethics Committee of *Santa Casa de Misericórdia de Goiânia Hospital* (SCMG) (CAAE:44025015.0.0000.5081). The clinical and research activities reported herein align with the Principles of the Declaration of Istanbul, as detailed in the “Declaration of Istanbul on Organ Trafficking and Transplant Tourism.” All participants provided informed consent in accordance with the committee requirements.

### 2.2. Volunteers

Between March 2016 and December 2017, 12 patients from the Kidney Transplant Unit at SCMG were chosen for participation by the Nefrovita Kidney Transplant Team.

The selection criteria for the participants were as follows:

Inclusion criteria:

1. Patients undergoing their first kidney transplant.
2. A minimum follow-up period of 12 months after transplantation.

Exclusion criteria:

1. Absence at 12-month follow-up.
2. Patients undergoing re-transplantation.
3. Declining participation in the study.

Blood samples were drawn into EDTA tubes before renal transplantation (T0). Subsequent collections were performed at 5 distinct intervals during the 12-month posttransplantation period T days (T7, T30, T90, T180, and T365). Additionally, 9 biopsies were sourced from two patients who exhibited graft rejection.

### 2.3. Enzyme immunoassay

Soluble HLA-G in patient plasma was quantified using the enzyme-linked immunosorbent assay (ELISA) method, as validated by Rebmann et al,^[15]^ which was carried out at the Molecular Biology Laboratory of the Medicine Faculty, Ribeirão Preto, São Paulo (FMRP/USP-RP), under the guidance of Dr Eduardo Donadi. The primary antibody used was anti-HLA-G MEM-G/9 (Exbio, Prague, Czech Republic), at a dilution of 1:100. This antibody identifies the shed HLA-G1 and soluble HLA-G5 isoforms. The secondary antibody was rabbit anti-human β2-microglobulin (Dako, Denmark) at a dilution 1:10,000. After each reaction step, the samples were washed four times using PBS 1× and 0.1% Tween 20 (Sigma, Saint Louis, MO). Absorbance was measured using a spectrophotometer at 450 nm.

To ascertain sHLA-G concentrations, we used a standard 5-point calibration curve (ranging from 6.25 to 100 ng/mL) with predetermined HLA-G5 levels. HLA-G5 was extracted from the supernatant of M8 melanoma cells transfected with the HLA-G pcDNA gene. The supernatant from M8 cells transfected with an empty pcDNA vector was the negative control. All tests were duplicated, and the results were expressed in ng/mL. To quantify the PD-L1 molecule concentration, we employed the MyBioSource kit (MyBioSource, San Diego, CA), according to the manufacturer’s instructions. The kit has a detection range of 2.25 to 36 ng/L, with a minimum detectable value of 0.1 ng/L, and all samples were assessed in duplicate.

### 2.4. Immunohistochemistry

Immunohistochemistry (IHC) was performed on biopsy samples from transplant recipients who exhibited graft rejection. For molecular detection, the MACH4 Universal HRP Polymer + DAB kit (Biocare, Concord, CA) was used according to the manufacturer’s guidelines. The antibodies used were anti-HLA-G MEM-G/2 (monoclonal antibody, Exbio, Prague, Czech Republic) and anti-PD-L1 (MyBioSource, San Diego, CA). Trophoblast biopsies served as the positive control for both antibodies, whereas the negative control entailed a duplicate sample without primary antibody pre-incubation.

The IHC results were interpreted by 2 independent pathologists in a double-blind manner. The evaluation was based on the presence or absence of immunostaining as well as its intensity. The intensity scale was delineated as negative (no staining, 0), weakly positive (+/++), or moderate/strong (+++/++++). A comprehensive examination of all sections was performed using a light microscope, focusing on high-power fields at 400× magnification.^[16]^

### 2.5. Statistical analysis

To assess the association between HLA-G and PD-L1 expression and categorical variables, Mann–Whitney or Kruskal–Wallis tests were employed, contingent on the number of subcategories. The Spearman correlation test^[17]^ was used to evaluate the relationship between numerical variables of interest. The Log-Linear Gamma Regression model^[18]^ was used to identify the factors linked to sHLA-G and PD-L1 expression. The generalized estimating equation (GEE) method^[19]^ was applied to recognize variables associated with sequential HLA-G and PD-L1 measurements in the same patients, the Generalized Estimating Equations (GEE) method^[19]^ was applied. All analyses were conducted using the R software (R Foundation for Statistical Computing, Vienna, Austria, 2020, Version 3.5.0). A *P*-value < .05 was considered significant.

## 3. Results

### 3.1. Clinical and laboratory findings

**Figure 1.**
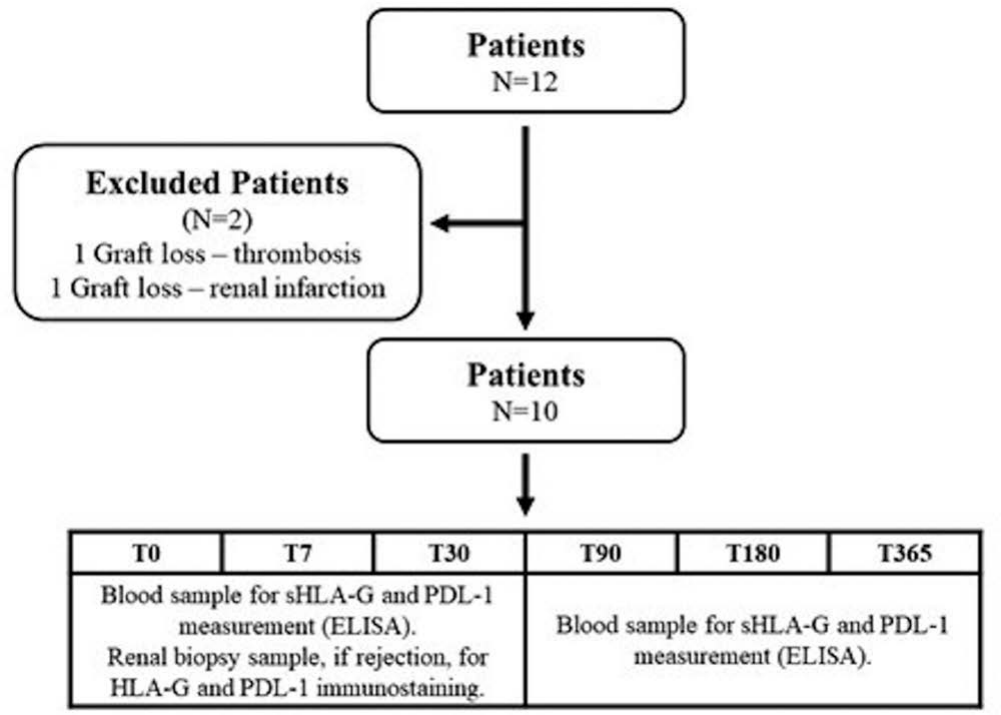
Flow chart illustrating patient enrollment in the study. ELISA = enzyme linked immunonosorbent assay, PDL-1 = programmed cell death ligand 1, N = number of patients, sHLA-G = HLA-G is a human leukocyte antigen G, restrictively expressed with suppressive functions, T0 = pre-renal transplant period, T7, T30, T90, T180, and T365 = days posttransplant.

Initially, the study followed 12 patients as per the protocol, from the pretransplant phase (prior to commencing immunosuppression) to 365 days posttransplant. However, during the study, 2 patients experienced graft loss (attributed to renal infarction and thrombosis). Consequently, the final analysis encompassed 10 patients observed over a 1-year follow-up period (Fig. 1).

Clinical and demographic evaluations indicated an average age of 40.83 ± 11.69 years among the participants. The majority of patients were male (83.3%), and glomerulonephritis emerged as the predominant diagnosis, accounting for 41.6% of the cases. Transplants primarily involved deceased donors (83.3%; Table 1.

**Table 1:** Clinical and demographic data of kidney transplant patients.

Variables	N	%
Sex		
Male	10	83.33
Female	2	16.67
Underlying kidney disease		
DM	1	8.33
ADPKD	2	16.67
GN	5	41.67
SAH	1	8.33
INDET	3	25.00
Type of transplant		
DD	10	83.33
RLD	2	16.67
Mismatch		
0–3	5	41.67
4–6	7	58.33
Cold ischemia time (hr)		
>15–24	6	60.00
>24	4	40.00
Delayed graft function		
No	3	25.00
Yes	9	75.00
Induction		
No	9	75.00
Yes	3	25.00
Bx-Rejection episodes		
1	2	16.67
2	1	8.33
None	9	75.00
Rejection type		
ACR	2	66.67
AMR	1	33.33
Blood pre-KT transfusion		
1–5	5	41.67
6–10	1	8.33
None	6	50.00
Pregnancy		
≥4	1	50.00
None	1	50.00

ACR = acute cell rejection, ADPKD = autosomal dominant polycystic kidney disease, AMR = antibody-mediated rejection, Bx-Rejection episodes = biopsy-confirmed rejection episodes, DD = deceased donor, DM = diabetes mellitus, GN = glomerulonephritis, INDET = indeterminate, KT = kidney transplant, RLD = related living, SAH = systemic arterial hypertension.

The cold ischemia duration ranged between 15 and 24 hours in 60% of the cases. Delayed graft function was observed in 9 patients (75%). The biopsy-confirmed rejection rate was 25%, with acute cellular rejection detected in 66.67% of the rejection incidents. Two renal graft losses (16.67%) were reported, both of which stemmed from vascular complications. Approximately 41.67% of patients received 1 to 5 blood transfusions. Notably, there was a consistent decline in the serum creatinine levels during the observation period. At the outset (T0), the average creatinine level was 8.17 mg/dL, which was reduced to 1.75 mg/dL by T365. Concurrently, the glomerular filtration rate saw an increase, averaging 51.15 ml/min at T365 (as seen in Table 1).

### 3.2. HLA-G plasma expression

Over the 12-month follow-up period, we observed a 67.4% reduction in sHLA-G expression from T0 to T30. Between T30 and T90, the expression levels increased; however, by T365, the average expression was lower than that at T0. A clear association was observed between time and HLA-G levels. As time progressed, there was a significant decrease in expression compared with the initial T0 measurements (Table 2).

**Table 2:** Multivariate variables associated with HLA-G dosage.

Variables	Initial model			Final model		
	Exp{*β*}	E.P. (*β*)	*P* value	Exp{β}	E.P. (*β*)	*P* value
Female	1.000	–	–	1.000	–	–
Male	2.155	0.21	<.001	1.814	0.18	.001
Type of KT – DD	1.000	–	–	1.000	–	–
Type of KT – RLD	3.004	0.00	<.001	2.425	0.13	<.001
Bx Ep. rejection – None	1.000	–	–	1.000	–	–
Bx Ep. rejection – 1	0.742	0.22	<.001	0.631	0.11	<.001
Bx Ep. rejection – 2	0.605	0.31	<.001	0.550	0.11	<.001
Pretransplant transfusion – None	1.000	–	–			
Pretransplant transfusion – 1 to 5	1.064	0.21	.763			
Pretransplant transfusion – 6 to 10	2.143	0.19	<.001			
T0	1.000	–	–	1.000	–	–
T7	0.657	0.29	.0082	0.644	0.26	.076
T30	0.333	0.17	<.001	0.326	0.11	<.001
T90	0.472	0.26	.006	0.440	0.27	.002
T180	0.295	0.31	<.001	0.289	0.22	<.001
T365	0.284	0.29	<.001	0.295	0.32	<.001

Bx EP = episode verified by renal biopsy, DD = deceased donor, HLA-G = human leucocyte antigen-G, KT = kidney transplant, RLD = related living donor; Transfusion = red blood cell concentration (300 mL), T0 = time before kidney transplant, T30, T90, T180, T365 = post kidney transplant time in days.

**Figure 2.**
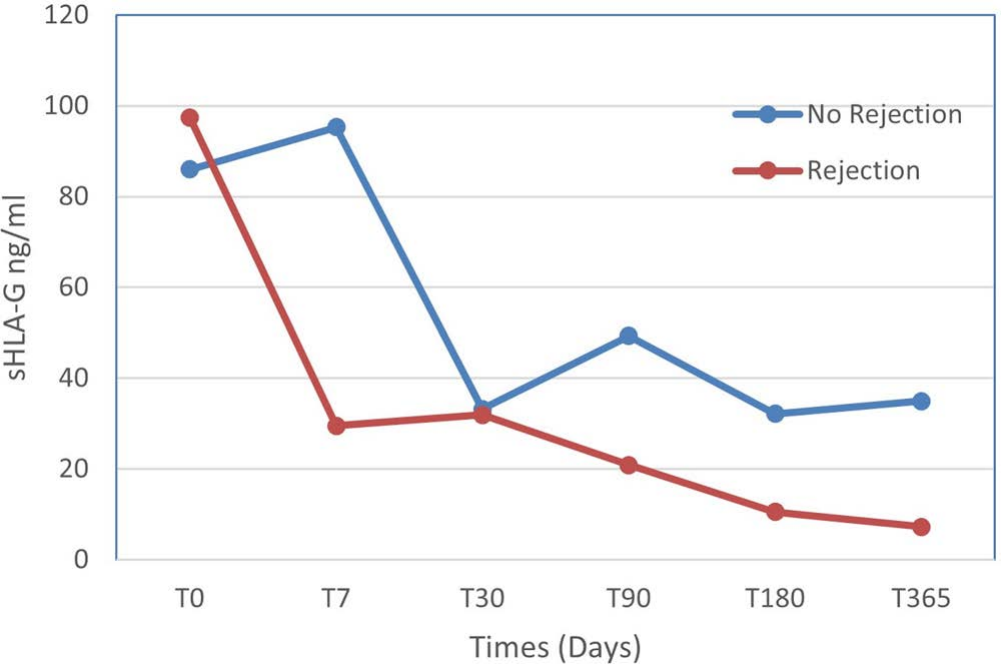
Comparison of average sHLA-G levels in patients with and without rejection. sHLA-G levels were assessed before the transplant (T0) and at 7, 30, 90, 180, and 365 days posttransplant. The red line represents levels in patients with rejection (T0: 97.37 ± 31.73; T7: 29.48 ± 17.93; T30: 31.88 ± 6.72; T90: 20.90 ± 4.31; T180: 10.54 ± 9.66; T365: 7.24 ± 2.59) whereas the blue line indicates levels in patients without rejection (T0: 85.98 ± 41.96; T7: 95.27 ± 11.27; T90: 49.33 ± 30.96; T180: 32.14 ± 16.01; T365: 34.97 ± 29.89). HLA-G is a human leukocyte antigen G, restrictively expressed with suppressive functions.

Low HLA-G expression is notably linked to rejection episodes, as confirmed by biopsy. Patients experiencing 2 rejection episodes exhibited a 45% reduction in molecular expression (*P < *.001) compared to those without any rejection episodes (Fig. 2). Additionally, patients who received kidneys from living donors had HLA-G levels 142.5% higher (*P* < .001) than those who received organs from deceased donors (according to Table 2).

### 3.3. PD-L1 expression

When evaluating PD-L1 expression, a clear correlation was observed between the underlying kidney diseases of the patient. Specifically, patients with diabetes mellitus exhibited a 578.8% increase in PD-L1 expression. In contrast, those with glomerulonephritis and dominant polycystic kidney disease displayed a decrease in PD-L1 expression by 41.7% and 55.5%, respectively, compared to patients whose kidney disease was related to systemic arterial hypertension.

The duration of cold ischemia also had a notable influence on PD-L1 expression. Specifically, ischemia lasting > 24 hour led to a 72.7% reduction in PD-L1 molecular levels.

**Figure 3.**
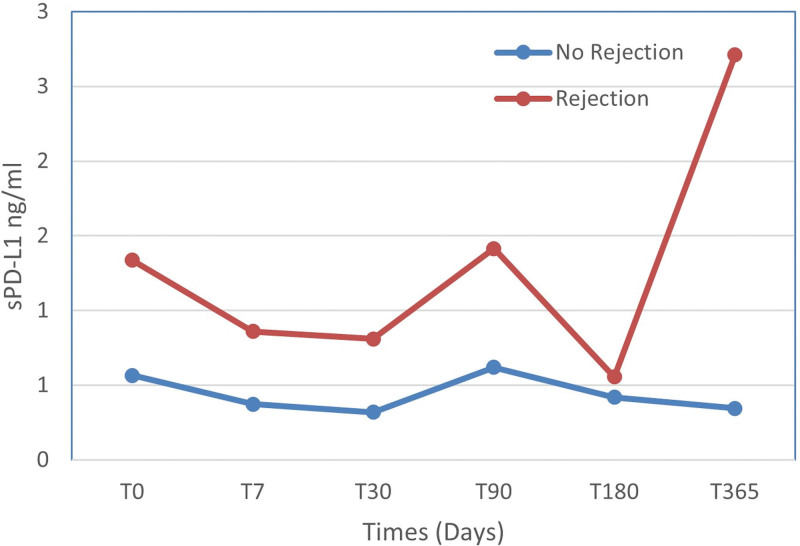
Comparison of average sPD-L1 levels in patients with and without rejection. sPD-L1 levels were assessed before the transplant (T0) and subsequently at 7, 30, 90, 180, and 365 days posttransplant. The red line represents levels in patients with rejection (T0: 1.34 ± 0.32; T7: 0.86 ± 0.74; T30: 0.81 ± 1.14; T90: 1.42 ± 2.00; T180: 0.56 ± 0.78; T365: 2.71 ± 0.37) whereas the blue line indicates levels in patients without rejection (T0: 0.57 ± 0.50; T7: 0.37 ± 0.27; T30: 0.32 ± 0.21; T90: 0.62 ± 0.94; T180: 0.42 ± 0.33; T365: 0.35 ± 0.24). PDL-1 = programmed cell death ligand 1.

Furthermore, the choice of immunosuppression protocol significantly affected the PD-L1 levels. Patients treated with a combination of corticosteroid, mTOR inhibitor, and calcineurin inhibitor experienced an 838.5% increase in PD-L1 expression. However, those on a regimen of corticosteroid + mTOR inhibitor + antiproliferative showed a 39.8% decrease in expression compared to those administered corticosteroid + calcineurin inhibitor + antiproliferative. Additionally, patients receiving 1 to 5 transfusions prior to transplantation demonstrated an 81.5% decrease in PD-L1 expression, while those receiving 6 to 10 transfusions showed a 64.7% reduction, compared to patients who did not receive transfusions (Table 3). For patients with 2 rejection episodes, PD-L1 levels were associated with statistical significance by multivariate analysis (as seen in Table 3 and Fig. 3).

**Table 3:** Multivariate variables associated with PD-L1 dosage.

Variables	Initial model			Final model		
	Exp (*β*)	E.P.	*P* value	Exp (*β*)	E.P.	*P* value
Male	1.000	–	–	1.000	–	–
Female	2.925	0.19	<.001	4.572	0.00	<.001
Bx Ep. rejection – None	1.000	–	–	1.000	–	–
Bx Ep. rejection – 1	3.525	0.22	.178			
Bx Ep. rejection – 2	1.735	0.33	.07		0	<.001
CMV infection – No	1.000	–	–			
CMV infection – Yes	0.532	0.24	.009	0.333	0.33	<.001
Pretransplant transfusion – None	1.000	–	–	1.000	–	–
Pretransplant transfusion – 1–5	0.343	0.26	<.001	0.185	0.63	.005
Pretransplant transfusion – 6–10	0.514	0.32	.033	0.353	0.57	.005
T0	1.000	–	–			
T7	0.722	0.18	.632			
T30	1.522	0.55	.355			
T90	4.179	0.29	.634			
T180	0.880	0.23	.150			
T365	0.811	0.80	.057			

Bx EP = episode verified by renal biopsy, CMV = cytomegalovirus, PD-L1 = programmed death 1 ligand, Transfusion = red blood cell concentration (300 mL), T0 = time before kidney transplant, T30, T90, T180, T365 = post kidney transplant time in days.

### 3.4. Immunohistochemistry

**Figure 4.**
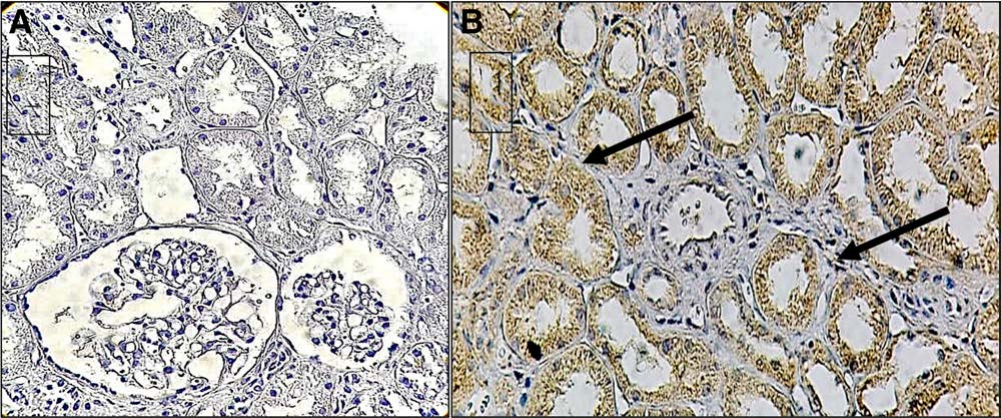
Renal tissue immunohistochemistry. (A) PD-L1: negative parenchyma and stroma. (B) HLA-G: granular cytoplasmic staining of renal tubules (arrows). Vascular wall and interstitium not marked. PDL-1 = programmed cell death ligand 1, sHLA-G = HLA-G is a human leukocyte antigen G, restrictively expressed with suppressive functions.

We observed granular heterogeneous immunostaining for HLA-G in all the assessed slides, with intensity variations ranging from 1 + to 4+. This staining was predominantly observed in the cytoplasm of renal tubules (as seen in Fig. 4). Neither the vascular wall nor the interstice showed immunostaining. Among the evaluated samples, 44.44% of the cells were moderately labeled. The average percentage of HLA-G + cells in renal tissue was 62.78% (Table 4). As for PD-L1 labeling, no expression was identified (Fig. 4). Satisfactory staining of PD-L1 positive controls confirmed that the methodology was appropriate. Subsequently, immunohistochemical reactions were performed on the same samples using the anti-PD-L1 antibody. In the present study, intense staining was observed.

**Table 4:** Renal tissue immunohistochemistry HLA-G

Biopsy sample	% marked cells	Intensity*	Observations
*P1B1*	50	1	Staining in tubular cells/fibroblasts, vascular endotheliosis
*P1B2*	60	2	Staining in tubular cells/fibroblasts, vascular endotheliosis
*P6B1*	90	3	Staining tubular, glomerular capillary endothelial cells
*P8B1*	50	1	Heterogeneous marking, tubular marking
*P9B1*	60	2	Marking of medullary and cortical tubules, absence of glomerulus
*P10B1*	90	1	Marked interstitial inflammatory infiltrate, tubular marking
*P12B1*	30	1	Tubular heterogeneous marking
*P12B2*	60	3	Tubular marking

B = biopsy sample, HLA-G = human leucocyte antigen-G, P = patient.

* Immunolabeling: intensity scale – negative (no staining, 0), weakly positive (+/++) or moderate/strong (+++/++++). Biopsies were only performed when the patient had an indication for kidney graft dysfunction.

## 4. Discussion

In our study, we evaluated HLA-G and PD-L1 expression behavior in transplant patients from the pretransplant stage to 365 days post-surgery. The correlation between the clinical and laboratory findings and the expression of both molecules was examined.

There was a notable decline in HLA-G levels. Throughout the 12-month study period, HLA-G expression levels remained below the initial measurement (T0). A similar observation was made by Piancatelli et al^[20]^ who assessed 103 patients both pretransplant and one year posttransplant.

Multivariate analysis revealed a significant correlation between certain factors and elevated HLA-G levels. Notably, patients who received organs from living donors showed increased HLA-G levels. This could be attributed to the minimal ischemia time during transplantation from a living donor, which mitigates ischemia-reperfusion injury. Furthermore, enhanced donor-recipient compatibility in these cases likely reduces the risk of acute rejection episodes.^[21,22]^

Conversely, an increase in rejection episodes and time correlated with diminished HLA-G expression. Parallel findings regarding rejection episodes were reported by Crispim et al and Jin et al^[23,24]^ Their research indicated that patients who underwent rejection episodes exhibited decreased sHLA-G levels. In our study, patients with one or two biopsy-confirmed rejection episodes had lower sHLA-G levels than those without any rejection. Xiao et al^[25]^ assessed the viability of sHLA-G5 as a posttransplant prognostic biomarker in 215 kidney transplant recipients. They determined that sHLA-G5 levels could predict acute rejection episodes with a sensitivity of 63.6% and a specificity of 82.1%. These findings highlight the potential of HLA-G as a prognostic marker for transplantation.

Regarding the variable “time,” it had a significant effect, causing a reduction across the evaluated durations. Krongvorakul et al^[26]^ explored the correlation between sHLA-G levels and allograft rejection immediately after transplantation. In a cohort of 76 kidney transplant patients, serum samples were collected on the 3^rd^ and 7^th^ post-operative days. They found no significant difference in the sHLA-G levels between patients with and without acute rejection during the assessment period. Similarly, in our study, T7 reading was not significantly related to HLA-G levels, mirroring the findings of Krongvorakul et al^[26]^

Poláková et al^[27]^ observed a decrease in HLA-G shortly after surgery (1–2 weeks), but observed a significant increase in patients without rejection from 1 to 12 months. In contrast, we observed a prominent reduction in HLA-G levels after T30. The discrepancies between the studies might be attributed to the clinical profiles of the patient groups and the sample size.

For PD-L1, there was a significant association between the number of pretransplant transfusions and its expression: a higher transfusion count corresponded to a lower PD-L1 level. This observation introduces a novel perspective on PD-L1 expression in kidney transplantation, with no current literature supporting this.

Regarding biopsy evaluation, the limited sample size precluded multivariate analysis, allowing only a descriptive assessment. HLA-G was highly expressed in more than half of the biopsy samples. Crispim et al^[23]^ detected HLA-G in 54.8% of biopsy samples, while Okushi et al^[28]^ employed immunofluorescence and noted HLA-G expression in 34.37% of the samples. Considering these findings, the expression of HLA-G in our samples was significant.

Future prospective cohort studies and clinical trials focusing on a “global” evaluation of immunologic risk related to HLA and non-HLA-related injury and immune monitoring using T cell receptors and other novel measures may provide much-needed insight into this complex research field. Integration of these findings into clinical practice may inform future personalized immunosuppression strategies, reduce the risk of posttransplant alloimmune responses, and prolong allograft survival and clinical outcomes following kidney transplantation.

### 4.1. Limitations

A small number of participants were included in the cohort, and the data presented are only from one transplant institution. In addition to the high cost, the research was more difficult to conduct, especially the long-running design, administrative changes, and funding difficulties.

In conclusion, our results underscore the link between HLA-G and PD-L1 expression, and prognostic indicators in kidney transplantation. Given the essential tolerogenic function of both HLA-G and PD-L1, investigations that track the expression dynamics of these molecules posttransplantation are crucial for their potential clinical use as prognostic biomarkers.

## Acknowledgments

We thank the patients, physicians, and professors involved in this study. Special Acknowledgments is given to Iocyene Silva for her invaluable assistance.

## Author contributions

**Conceptualization:** Silvia M. Botelho, Isabela J. Wastowski, Antonio da Silva Menezes Jr.

**Data curation:** Silvia M. Botelho, Isabela J. Wastowski, Antonio da Silva Menezes Jr, Renata T. Simões.

**Formal analysis:** Silvia M. Botelho, Isabela J. Wastoski, Renata T. Simões.

Antonio da Silva Menezes Jr.

**Funding acquisition:** Silvia M. Botelho, Isabela J. Wastowski, Antonio da Silva Menezes Jr.

**Investigation:** Silvia M. Botelho, Isabela J. Wastowski.

**Methodology:** Silvia M. Botelho, Isabela J. Wastowski, Renata T. Simões, Maria A. P. C. Cysneiros, Nílzio A. da Silva.

**Project administration:** Silvia M. Botelho, Antonio da Silva Menezes Jr.

**Resources:** Silvia M. Botelho, Isabela J. Wastowski, Antonio da Silva Menezes Jr, Aline L. Rezende.

**Software:** Silvia M. Botelho, Isabela J. Wastowski, Antonio da Silva Menezes Jr.

**Supervision:** Silvia M. Botelho, Isabela J. Wastowski, Renata T. Simões, Antonio da Silva Menezes Jr, Nílzio A. da Silva.

**Validation:** Silvia M. Botelho, Renata T. Simões, Maria A. P. C. Cysneiros, Antonio da Silva Menezes Jr, Aline L. Rezende, Nílzio A. da Silva.

**Visualization:** Silvia M. Botelho, Isabela J. Wastowski, Antonio da Silva Menezes Jr, Nílzio A. da Silva.

**Writing—original draft:** Silvia M. Botelho, Isabela J. Wastowski, Renata T. Simões.

**Writing—review & editing:** Silvia M. Botelho, Isabela J. Wastowski, Antonio da Silva Menezes Jr.
